# Public involvement in big data projects: an ethnographically-informed study.

**DOI:** 10.23889/ijpds.v7i3.1992

**Published:** 2022-08-25

**Authors:** Elizabeth Nelson, Dermot O'Reilly

**Affiliations:** 1 Administrative Data Research Centre Northern Ireland

The road to policy-engaged research in Northern Ireland has not been smooth. In January 2017 the devolved government at the Stormont Assembly collapsed and devolution was not restored until January 2020. Political turmoil resurfaced in February 2022, with the collapse of the Executive. Trying to engage with and influence policy during these periods has been difficult, but necessary to achieve research impact and demonstrate the utility of data access for research purposes, thus supporting the acquisition of further datasets. This required a paradigm shift in the way research programmes were conceived of and delivered; i.e. away from largely curiosity-based research for academic interest and benefit and towards one that necessitated a more direct engagement and involvement of the government departments and data owners who would benefit from better evidence generated by administrative data research, and incorporate this evidence into their policymaking and service provision.

Aligning the academic and policy agenda increased the likelihood of successful data acquisition, the prerequisite for research success and subsequent research impact. It also creates a more stable environment for data research to flourish, even in challenging political circumstances.

This presentation will detail the approach developed by researchers and engagement professionals in Northern Ireland during political and governmental collapse to embed and enhance the potential impact that data research can have on policy by supporting the design and development of research that answers the questions most relevant to policymakers whilst also maintaining academic independence and integrity. It will offer suggestions for engaging and involving political representatives and policymakers, and for creating a resilient, robust and sustainable approach to policy impact that can withstand shifting political sands, based on recognised best practice of embedded engagement built to develop and maintain trust.

